# The Impact of the Digital Home Environment on Kindergartners’ Language and Early Literacy

**DOI:** 10.3389/fpsyg.2020.538584

**Published:** 2020-09-18

**Authors:** Eliane Segers, Tijs Kleemans

**Affiliations:** ^1^Behavioural Science Institute, Radboud University, Nijmegen, Netherlands; ^2^Department of Instructional Technology, University of Twente, Enschede, Netherlands

**Keywords:** home literacy environment, kindergarten, digital home environment, early literacy, parental expectations

## Abstract

We examined whether a digital home literacy environment could be distinguished from a (traditional) analog home literacy environment, and whether both were related to kindergartners’ language and literacy levels, taking parental expectations into account. Caregivers of 71 kindergarteners filled out a questionnaire on the home environment (expectations, activities, and materials), and the children were assessed on language (vocabulary and grammar) and literacy (begin phoneme awareness, segmentation skill, and grapheme knowledge) skills. Results showed that a digital environment could be distinguished from an analog environment. However, only the analog environment was related to children’s language abilities. Parental expectations were related directly to both language and literacy abilities. The fact that there was no relation between the digital home environment and language and literacy outcomes might indicate large variation in the quality of the digital home environment. More attention is needed to this part of daily life when growing up in a digital society.

## Introduction

During their kindergarten years, young children increasingly become aware of language and literacy. They enter kindergarten with heads full of stories that have been told at home and an emergent awareness of the form and function of written language. During their kindergarten years, children have a steep growth in the development of vocabulary (Biemiller, 2006), and also begin to develop phonological awareness and grapheme knowledge (e.g., Verhoeven et al., 2016). The home literacy environment is an important factor in this development, as evidenced by a large body of literature described in meta-analyses by Bus et al. (1995) and more recently by Mol and Bus (2011). The home literacy environment has experienced a sudden shift with the introduction of the tablet computer. Tablet computers entered households in 2010 and, in contrast to the personal computer, became much more a device that young children could easily use and were also allowed to use (Plowman and McPake, 2013). Not only many apps are available for use on tablets, including e-book reading apps, but also apps that focus on early literacy. While there is a large body of research on the additional effects of computer-supported early literacy in kindergarten (see Verhoeven et al., 2020), only very recently has research been published on the use of tablets at home by kindergartners. These studies show positive relations between tablet use at home and early literacy (Neumann, 2016). However, research is lagging behind on the impact of a digital home environment on learning (Radesky et al., 2015). In fact, it is unclear to what extent an actual *digital home literacy environment* (DHLE) can be distinguished from what we will call an *analog home environment*, and whether such a digital environment further adds to children’s language and early literacy.

### The Analog Home Literacy Environment

The (analog) home literacy environment, often described as the shared literacy activities between parents and their children (van Steensel, 2006), accounts for a substantial amount of the variation in the development of language and early literacy (see e.g., Bus et al., 1995; Mol and Bus, 2011). Various facets of the home literacy environment have been studied, such as frequency or amount of parental book reading and shared book reading. Burgess et al. (2002) made clear that the home literacy environment should be studied as a broader concept, for example, by including singing and playing language games or engaging in letter-based activities.

In a landmark study by Sénéchal et al. (1998), it was shown that storybook exposure is mostly related to oral language development (i.e., vocabulary, listening comprehension, and phoneme awareness), while parental teaching predicts written language skills (e.g., knowledge of the alphabet). Burgess et al. (2002) also showed that especially parental activities aimed to engage their child in literacy were predictive of early literacy development.

Following up on these results, Sénéchal and colleagues (e.g., Sénéchal and LeFevre, 2002; Sénéchal, 2006) proposed a Home Literacy Model that distinguishes between informal (e.g., storybook reading) and formal (e.g., parental teaching) literacy activities. Their research again showed that the informal literacy activities in general are predictive of oral language, but not early literacy, while formal literacy activities predict early literacy, but not oral language. These results were recently replicated in a transparent orthography (Finnish), albeit that effects of maternal teaching were smaller (Silinskas et al., 2020).

Along with parent-child literacy activities, the home literacy environment also consists of experiences in which children explore print on their own (see Sénéchal et al., 2017). However, this aspect has often not been taken into consideration in questionnaires, as the focus has mostly been on parent-child interactions.

In addition to activities, parental beliefs and expectations about their children have a major impact on the home literacy environment. Martini and Sénéchal (2012) showed how both beliefs and expectations had a direct and an indirect effect *via* formal literacy activities on early literacy. In a similar vein, Davis-Kean (2005) showed, in a large longitudinal study, how parental expectations impacted parental (reading) behaviors, which in turn impacted academic achievement in 8–12-year-olds. Again, parental expectations had a strong indirect effect on children’s achievement. Also, Silinskas et al. (2020) showed that maternal beliefs and expectations were positively related to formal literacy activities, and not so much to informal literacy activities.

### The Digital Home Literacy Environment

The DHLE can be described as the shared literacy activities between parents and children while using a digital device, and the time children spend playing with such a device on their own. Many Western households nowadays have more than one tablet at home (also including smartphones; MarketingCharts, n.d.), and young children are often allowed to play on them (Plowman and McPake, 2013) or even have one of their own. Holloway et al. (2013) reported that tablet use in young children is growing as well. For example, 50% of Swedish children aged between 3 and 4 use tablet computers, and these numbers are growing across countries, and they are related to parental use of devices. The development of apps for the tablets is a huge industry, and there are many early literacy tablet-apps available in online stores. In a recent study on media use of young children in Australia, Huber et al. (2018) reported that preschoolers have about 80 min of screen time per day, which increased to almost 100 min for school-aged children. Time with a touchscreen seems dominated by watching videos, but also time was spent playing (educational) games. The general role of access to media was studied by Liebeskind et al. (2014). Their results showed little effects of amount of media in the households (e.g., number of computers at home) on language skills of young children (8–36 months). This study did not specifically address tablets or questions about parent-child activities using different media. Parents in the Huber et al. (2018) study reported strong agreement on the potential of technology as a learning tool. Parents tend to have device restrictions to prevent their child to spend too much time with a device, but are also actively involved in their young child’s media use (Zaman et al., 2016).

Besides the obvious disadvantages of spending too much time with a tablet, e.g., when using it as a television, and passively watching movies, a world of possibilities has opened regarding home literacy activities. Apps are available that provide digital story books, which have been shown to benefit language development (Ihmeideh, 2014; Takacs et al., 2015). In a similar vein, apps that provide games on phonological awareness of grapheme-phoneme correspondences can boost early literacy (see Verhoeven et al., 2020), and as such may provide an additional effect over and above the traditional/analog home literacy environment (AHLE) specifically regarding early literacy skills. Research has just begun to examine what children can learn from tablet apps. In a pioneering study on this topic, Neumann (2016) showed how home tablet activities of the child correlated with emergent literacy measures in 2–4-year olds. Interestingly, she did not ask about joined parent-child tablet activities. In this study, tablet writing related to print awareness, print knowledge, and sound knowledge, but Neumann did not study whether digital activities predicted emergent literacy over and above non-digital literacy activities, or related the tablet measures to analog (i.e., non-digital) home literacy measures.

Herodotou (2018) is probably the first to have written a review on the effects of tablets on learning and development of young children (2–5-year olds). Herodotou identified five (quasi)-experimental and four descriptive studies on the effects of touch screen tablets on early literacy, but did not include the extensive literature on digital books (Bus et al., 2015). She concluded that effects of tablet use by young children were found mostly on vocabulary and print knowledge. Most studies were conducted with parents and children in a joint activity, and not necessarily aiming to compare tablet vs. traditional print, which leaves the question unanswered whether the use of tablets as a digital activity can be distinguished from, and adds to, children’s analog home literacy experiences.

Neumann (2018) studied the scaffolding role of the parent in young children’s tablet use, but did not relate this to learning outcomes in language and literacy skills. Kim and Anderson (2008), however, showed that mother-child interactions tended to be more complex in electronic context vs. traditional print format. Furthermore, Teepe et al. (2017) showed that technology-enhanced storytelling had a positive effect on children’s vocabulary skills in a pretest-posttest control condition. However, it has also been shown that digital storybooks can be distracting. Krcmar and Cingel (2014), for example, compared parent-child book reading on an iPad tablet vs. a traditional book. Children (2–5-year olds) had a better story comprehension in the traditional book condition, probably due to the fact that parents included more distractive talk in the digital condition.

### The Present Study

The home literacy environment is an important influencer of the development of children’s language and literacy development. So far, the literature has not made a distinction between a digital vs. an analog home-environment, and also in recent studies regarding the home literacy environment, the digital literacy environment was not included (e.g., Hamilton et al., 2016), while digital technology has invaded the lives of the children. In fact, it remains unclear whether the two can be distinguished empirically, and, if so, whether the DHLE adds to the explanation of language and early literacy in kindergartners. In households with more digital devices, children also use them at a younger age (Holloway et al., 2013). Meta-analyses have shown the possible (additional) benefits of apps focusing on language and literacy (Takacs et al., 2015; Verhoeven et al., 2020). Differences between households with a higher or lower digital literacy environment may thus emerge, and impact language and literacy development.

In the present study, the first research question, therefore, was: can a DHLE be distinguished from an AHLE? We expected to be able to distinguish between an analog and a DHLE.

The second research question was (a) what is the additional value of the digital home literacy environment on language and early literacy over and above parental expectations and the AHLE and (b) do both home environments mediate between parental expectations and children’s language and early literacy? We expected effects of the analog home environment to be especially visible regarding language skills, while the digital home environment might have a stronger (and additional) impact on early literacy, as children could be more confronted with exercises in literacy apps. We expected parental expectations to be related to language and early literacy, which would be partly mediated by both the analog and the digital home environment. Since the home literacy environment and children’s language and literacy outcomes are associated with intelligence and family’s SES (e.g., Pace et al., 2017), we took these into account.

## Materials and Methods

### Participants

Three schools with 11 mixed first‐ and second-year kindergarten classes in the southern part of the Netherlands took part in the study in spring 2017. In the Netherlands, kindergarten is a two-year program, prior to first grade. Teachers pay attention to emergent literacy, and storybook reading is common practice. Letters were sent to the parents of the second-year kindergartners; i.e., the group of children in the year prior to grade 1. Seventy-one parents gave informed consent for their child to participate and filled out the questionnaire. There were no specific exclusion criteria. However, one child was not included in the analysis for having too little knowledge of Dutch to understand the tasks. The average age of the remaining 70 children was 5; 11 (i.e. 5 years; 11 months; *SD* = 4.3 months). There were 34 boys and 36 girls in the sample.

The main caregiver was asked to fill out the questionnaire; this was done by 57 mothers and 12 fathers, while one parent did not fill in this question. The average age of the main caregiver was 37.65 years (*SD* = 5.25). The educational level was vocational or lower (three only primary education and four only secondary education) for 37 caregivers, the educational level was university of applied sciences or university for the remaining 33 caregivers. This variable was therefore dichotomized (0 = lower education level and 1 = higher educational level) in further analyses. In most households, Dutch was the main language; seven parents indicated that Dutch was hardly ever spoken at home, and one that it was only spoken a few times per week. In 25.7% of the families, another language was spoken: Chinese (*n* = 5), English (*n* = 2), Turkish (*n* = 7), Moroccan (*n* = 2), Spanish (*n* = 1), and Ghanaian (*n* = 1). This percentage is in line with the percentage of children with a migration background in current Dutch primary education (Central Bureau of Statistics, 2020).

### Materials

#### Child Factors

##### Non-Verbal Intelligence

As an indication of non-verbal intelligence, we used the subtest exclusion from the RAKIT-2 (Resing et al., 2012). The subtest consists of 65 items, with increasing difficulty. Each item consisted of four stimuli in which three of them belonged to the same rule(s). Children are asked each time which stimulus did not belong, for example, because of its shape. Testing was stopped after four mistakes in five consecutive items. The score comprised the number of correctly answered items. Reliability is good (Cronbach’s alpha = 0.88; Pieters et al., 2013).

##### Language and Early Literacy

Five tasks were administered to assess language and early literacy skills. First, *grammatical skills* were assessed using the subtasks Sentence Comprehension 1 and 2 from the Taaltoets Alle Kinderen (Language Test for All Children; Verhoeven and Vermeer, 2006). Knowledge of function words and conjunctions are the focus of these tasks. The tests contain 21 items each, preceded by two practice items. The child has to choose the correct drawing out of a series of three. An example is “The cat sits on the chair,” with pictures showing a cat on a chair, next to a chair, or under a chair. Cronbach’s alpha of 0.82 is reported. Furthermore, *vocabulary knowledge* was measured using the passive vocabulary test from the same Language Test (Verhoeven and Vermeer, 2006). Now, children had to choose the correct picture out of a series of four that matched the word pronounced by the experimenter. The test consists of 96 items, preceded by two practice items. The items increase in difficulty, and assessment is terminated after five consecutive mistakes. Cronbach’s alpha of 0.97 is reported. In addition, *begin phoneme awareness, segmentation skill*, and *grapheme knowledge* were assessed with tasks developed by Schaars et al. (2017). For begin *phoneme awareness*, the child is asked to isolate the first phoneme of a one-syllable word pronounced by the experimenter (e.g., say “/k/” when the experimenter says “cat”). The task consists of 10 items, preceded by two practice items (Cronbach’s alpha = 0.83; Schaars et al., 2017). For *segmentation skills*, the child has to pronounce all phonemes of each one-syllable word pronounced by the experimenter (e.g., say “d-o-g” when the experimenter says “dog”). The tasks consist of 10 items, preceded by two practice items. Cronbach’s alpha was 0.85 (Schaars et al., 2017), indicating good reliability. And finally, for *grapheme knowledge*, the child was presented with a card that contained the 34 graphemes that children learn in Dutch Grade 1 (including digraphs, such as “aa”). The child is asked to name the sound of all graphemes it knows. This task had excellent reliability (Cronbach’s alpha = 0.93; Schaars et al., 2017).

A principal component analysis on the five language and literacy measures that were assessed revealed two components, with 80.54% explained variance (see Table 1). All measures clearly loaded on one dimension, although *grammatical skills* also loaded >0.4 on the second dimension, however, with a clear preference for the first. We transferred the scores on each of the tasks to z-scores and added the two, respectively, three scores to a score for *language ability* and a score for *early literacy*.

**Table 1:** Structure matrix of the principal component analysis on language and early literacy.

Question	Component	
	Language skills	Early literacy
Vocabulary	0.928	
Grammar	0.881	0.427
Begin phoneme		0.896
Segmentation		0.884
Grapheme knowledge		0.853

#### Home Literacy Environment

The home literacy environment was measured with a questionnaire based on Segers et al. (2015) that, however, did not take the digital environment into account. The first part contained demographic background questions. Questions were asked who the primary caregiver was and what his/her age was. We also asked how often Dutch was spoken at home [on a 4-point scale, ranging from “(hardly) ever (1)” to “daily (4)”], which language was spoken at home, and what the level of parental education was (as a proxy for socio-economic status).

Regarding the home literacy questions, we made several modifications. First, each question was duplicated, asking whether an activity occurred in an analog manner and, next, whether it occurred digitally. In addition, we also asked about activities that the child did on his/her own, as this may be typical for digital activities. Furthermore, we added questions on the general home environment, regarding number of paper and digital books at home and the number of devices (PCs, tablets, and smartphones) at home. Part 2 asked questions about the availability of materials at home: number of paper and digital books, and number of devices (television, computer/laptop, tablet, smartphone, e-reader, music player, DVD-player, and gaming console). Part 3 asked five questions regarding frequency of both analog and digital parental activities with the child (a total of 10 items): reading to the child; stimulating the child to read; stimulating the child to write; playing language and word games; and singing/reading poems, songs, and rhymes. In a similar vein, four questions were asked about activities the child would conduct on its own both analog and digital formats (i.e., eight items): looking into (picture) books; letter naming; playing language and word games; and listening to poems, songs, and rhymes. The answers could be indicated on a 5-point scale ranging from “hardly ever (1)” to “several times a day (5).” Part 4 focused on math activities, which is not part of the current study. Part 5 asked about parental expectations regarding language, literacy, and numeracy, the latter not being part of the current study. Parental expectations focused on language and early literacy. Parents were asked in six questions to estimate whether, at the end of kindergarten, their child would be able to name all the letters, write his/her own name, rhyme, segment words, decode cvc-words, and retell a short story using a 4-point Likert scale ranging from “not at all (1)” to “good (4).”

### Procedure

After three schools were found that were willing to participate, all children from those schools who were in the second-year of kindergarten received an envelope from their teacher containing the questionnaire and consent form, following ethical guidelines of our research institute. They were asked to give this envelope to their parent. Filled out questionnaires could be returned to the schools in a sealed envelope and were collected by one of the test assistants. In total, 139 children were given an envelope. The parents of one child reported that they did not want their child to participate, while 67 parents did not respond (also not after receiving an e-mail from the school as a reminder). In total, 71 parents filled out the questionnaire and gave consent for their child to participate. This response rate is quite normal in this type of active consent procedure (Esbensen et al., 1999).

Next, children were tested in three sessions of no longer than 30 min on language and literacy measures in a quiet room inside the school. Each child had a maximum of two test sessions per day, always with at least a lunch break in-between. Early numeracy was also assessed in the first of these sessions, and working memory (being related to early numeracy) in the second, but these were not included in the current paper. In the second session, non-verbal intelligence and early literacy (except grapheme-phoneme knowledge) was assessed and in the final session, grammatical skills, vocabulary, and grapheme-phoneme knowledge were assessed. Sessions two and three were assessed in random order. Test assistants were six undergraduate and graduate students of educational science with experience in testing young children. Before seeing the children, the students received half-day training by the second author (an educational psychologist).

### Statistical Approach

To answer the first research question, we analyzed the data from the parental questionnaires. Questions that did not have a normal division (−1.5 < skewness and/or kurtosis > 1.5) were removed. Next, we ran the principal component analysis with direct oblimin rotation and inspected the scree plot as an indication for the number of components. The scores of the questions per component were summed up for the remaining analyses.

To answer the second research question, mediation analyses using the PROCESS add-on in SPSS (Hayes, 2013) were conducted. Parental expectations were the independent variable, digital and analog home environment were the mediators and language ability and early literacy, respectively, were the dependent variables. Boot-strapping was set at 5000 cycles, as recommended by Hayes. The mediation model was set up this way following the theoretical model described in the introduction. The total effect of parental expectations on the outcome measure is broken down into a direct effect and an indirect effect *via* the mediators.

## Results

### Preliminary Considerations

Within the 70 questionnaires that were returned by the parents, there was a relative high level missing answers in the questions regarding digital activities, even though a computer/tablet was reported to be at home and accessible to the child. The analyses in this Results section, therefore, often reflect the smaller number of respondents who filled out both analog and digital questions. A total of 15 out of the 70 questionnaires that were returned had missing values for the digital, but not the analog questions. Furthermore, two children had a missing score on one of the early literacy skills measures. The group of children of the parents who did not fill in the questions on digital home environment had lower language skills than the other group [*t*(68) = −2.18, *p* = 0.03, *d* = 0.62], but did not differ in early literacy or non-verbal intelligence (all *p* > 0.35). Of the 15, nine parents had a lower education and six parents had a higher educational level. In the remaining group, 28 had a lower education whereas 27 parents had a higher education. Of the six questions on parental expectations, one item was removed because of a high kurtosis (being able to write its own name). The remaining five items were summed up to reflect “parental expectations.” Reliability was good (Cronbach’s alpha = 0.846).

### Analog vs. Digital Home Literacy Environment

We first explored the data to find out whether an AHLE could be distinguished from a DHLE (i.e., the first research question). Four questions regarding DHLE did not have a normal division (−1.5 < skewness and/or kurtosis > 1.5) and were removed because of this [reading to child, stimulating child to read, stimulating child to write, and looking at (picture) books]. We ran the principal component analysis on the remaining 14 questions of the questionnaire, and inspected of the scree plot showed the point of inflection at three components. We thus reran the analysis forced on two components, which resulted in 62.78% explained variance. The structure matrix (see Table 2) indicates that a first component can be distinguished as the digital home environment, including five questions with a loading >0.7. Reliability was good (Cronbach’s alpha = 0.847). The second component that can be distinguished is the analog home environment with four remaining questions with a loading >0.6. Reliability was acceptable (Cronbach’s alpha = 0.702). Five questions loaded high on both factors and were not included, since they were not distinctive.

**Table 2:** Structure matrix of the principal component analysis on the home literacy questionnaire.

Question	Component	
	Digital home literacy environment (DHLE)	Analog home literacy environment (AHLE)
Digital together: poems and songs rhymes	0.885	
Digital alone: poems and songs, rhymes	0.882	
Digital together: language and word games	0.849	
Digital alone: language and word games	0.859	
Digital alone: letter naming	0.720	
Analog together: stimulating child to read		0.741
Analog alone: looking into (picture)books		0.708
Analog together: reading to child		0.691
Analog together: stimulating child to write		0.644
Analog together: language and word games	0.719	0.610
Analog alone: language and word games	0.610	0.500
Analog alone: letter naming	0.445	0.623
Analog together: poems and songs rhymes	0.622	0.715
Analog alone: poems and songs rhymes	0.606	0.652

### The Role of the Digital Home Environment

The second research question addressed the role of the digital home environment. Table 3 shows means and standard deviations of the variables under study. We checked *via* independent samples *t*-tests whether the (dichotomized) educational level of the main caregiver made a difference regarding the language ability or the level of early literacy of the child. This was not the case [language ability: *t*(68) = −0.63, *p* = 0.53, *d* = 0.15 and early literacy: *t*(66) = −0.93, *p* = 0.36, *d* = 0.22], and hence this variable was not taken into account in the remaining analyses, to retain statistical power.

**Table 3:** Descriptive statistics of home literacy environment (HLE) and child abilities. For language ability and early literacy, the factor scores are provided, as well as the sum scores for each subtest.

Variables	*n*	*M*	*SD*	Min	Max
Non-verbal intelligence	70	27.40	8.42	7	46
Language ability (factor score)	70	0	1.81	−4.31	2.86
Grammar	70	34.80	4.17	23	41
Vocabulary	70	64.53	13.42	33	85
Early literacy (factor score)	68	−0.01	2.65	−4.49	5.66
Begin phoneme	70	6.34	3.12	0	10
Synthesis	68	2.76	2.85	0	10
Grapheme knowledge	70	13.59	8.42	1	31
Number of books at home	69	2.94	0.95	1	4
Number of digital books at home	54	1.87	1.23	1	4
Number of devices at home	67	8.75	1.81	4	12
Analog HLE	70	12.51	3.29	5	20
Digital HLE	55	10.27	4.77	5	22
Parental expectations early literacy	68	15.56	3.49	7	20

Table 4 depicts the correlations between the different variables. As can be seen, traditional measures such as the number of books at home and the non-verbal intelligence are associated with language ability and early literacy, and so are the parental expectations. The table shows that the (traditional) analog home environment is associated with language ability, but not early literacy nor parental expectations. The digital home environment, however, is only associated with parental expectations. The general digital home environment (digital books at home and the number of devices at home) is only related to the number of (paper) books at home, but not to any of the child factors.

**Table 4:** Correlations between child abilities and home environment (Spearman), and home literacy environment (Pearson).

S. No.		1	2	3	4	5	6	7	8	9
1	Non-verbal IQ	-								
2	Language ability	0.30^*^	-							
3	Early literacy	0.49^**^	0.33^**^	-						
4	Number of books at home	0.30^*^	0.37^**^	−0.02	-					
5	Number of digital books at home	0.18	0.18	−0.05	0.27^*^	-				
6	Number of devices at home	0.14	0.08	0.09	0.17	0.20	-			
7	Analog HLE	0.27^*^	0.28^*^	0.20	0.19	0.08	0.05	-		
8	Digital HLE	0.04	0.22	0.18	−0.05	0.11	0.13	0.05	-	
9	Parental expectations	0.16	0.40^**^	0.65^**^	0.14	0.14	0.22	0.17	0.43^**^	-

* *p < 0.05*;

** *p < 0.01*.

The second research question was asked to examine the role of the digital home environment on both language ability and early literacy. While the correlation table already suggests that no direct effect of the digital home environment on language ability or early literacy will be found (i.e., research question 2a), the analyses might reveal a mediating effect (i.e., research question 2b).

Regarding *language ability*, the *R*^2^ of the final model of the mediation analysis was 0.21 (*p* = 0.01), with a sample size of *n* = 55. There was no significant effect from the digital home environment on language ability. Both parental expectations and the analog home environment predicted language ability, but there were no indirect effects [indirect effect *via* analog home environment 95% CI (−0.03–0.07) and indirect effect *via* digital home environment 95% CI (−0.04–0.09)]. Parental expectations were related to the digital, but not analog environment. When adding non-verbal intelligence as a covariate to the model (on the dependent variable), most of the variance was taken away by this measure. The effects of both the analog home environment and the expectations were no longer significant (*p* = 0.09 and *p* = 0.06, respectively), while only the effect of non-verbal intelligence was significant (B = 0.09, *p* = 0.003). Adding the number of books at home, instead of non-verbal intelligence led to a still significant model, but with none of the variables having a unique effect.

Regarding *early literacy*, the *R*^2^ of the final model of the mediation analysis was 0.42 (*p* < 0.01), with a sample size of *n* = 54. There were no significant direct or indirect effects from the analog or digital home environment on early literacy [indirect effect *via* analog home environment 95% CI (−0.01–0.06) and indirect effect *via* digital home environment 95% CI (−0.13–0.03)]. Only parental expectations predicted early literacy. Parental expectations were related to the digital, but not analog environment. The total effect of parental expectations on early literacy was 0.54 or 0.50 depending on which home environment was mediator or covariate on the dependent variable in the model. When adding non-verbal intelligence or the number of books at home as a covariate to the model (on the dependent variable), the effects remain similar, and neither non-verbal intelligence nor the number of books was a significant predictor of early literacy.

**Figure 1:**
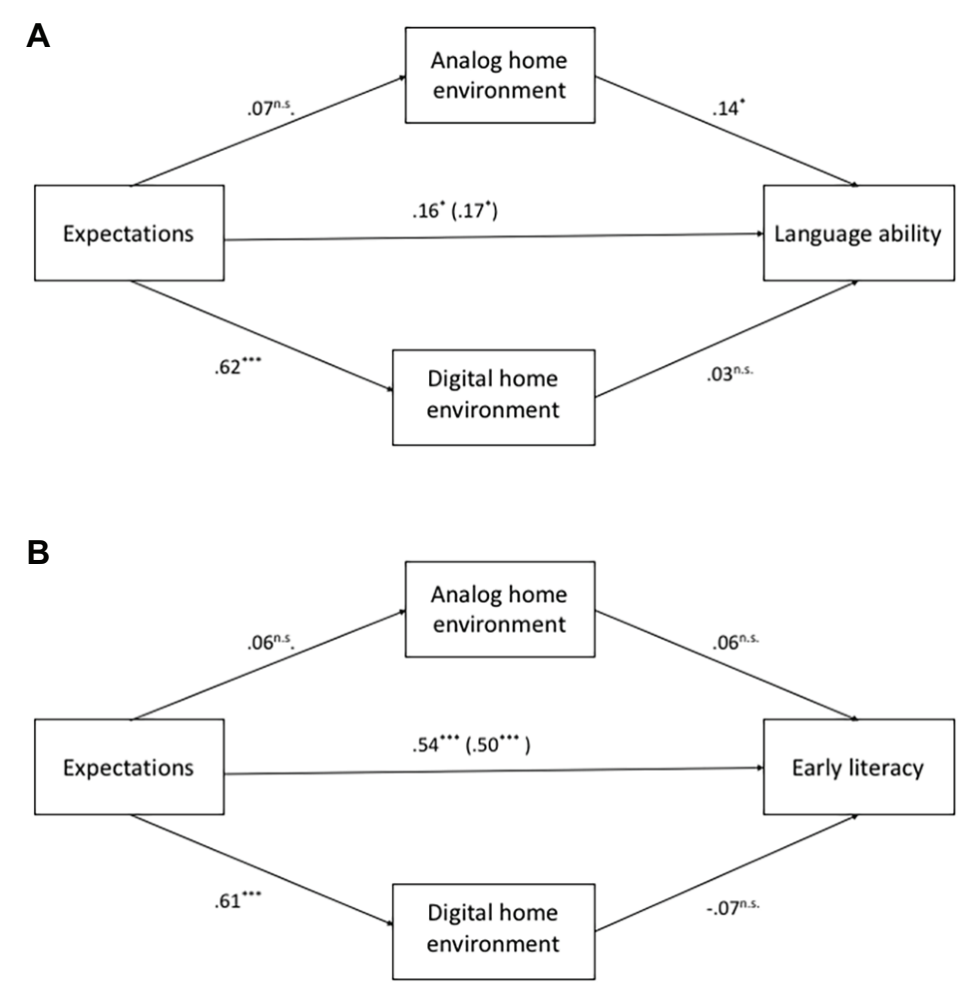
The mediating role of analog and DHLE in the relation between parental expectations and language ability **(A)** and early literacy **(B)**. ^*^p < 0.05; ^***^p < 0.001.

Figures 1A,B show the results.

## Discussion

The goal of the present study was to find out whether a DHLE could be discriminated from an analog home environment and, if so, whether this would have an additional impact on children’s language and early literacy. The results suggest that a DHLE can be seen as separate from an AHLE, but that only the latter is related to language ability. Parental expectations strongly related to both language ability and early literacy, but there was no indirect relation *via* parental activities.

Our first hypothesis was that we would be able to distinguish between an analog and a digital home environment, and we indeed found evidence for this. We had included questions on whether activities were carried out alone by the child or together with the parent. However, results did not show that the “alone” activities were the ones that were done digitally, and the “together” activities the ones that were done together. This indicates that children do not typically play alone with a digital device, while other language and literacy activities are done with their parents. In other words, digital is not the same as alone for these young children. This is in line with results reported by Huber et al. (2018). An easy way to think about the home environment is that a high-literacy environment is a beneficial environment, regardless. However, the current results suggest that the home environment cannot be seen as a general factor, and that there is variation in how literacy is addressed at home either analog or digital. The fact that the home environment as such cannot be seen as a general environment is in line with results from Segers et al. (2015) who showed that a literacy environment can be distinguished from a numeracy environment with unique predicting value on literacy and numeracy.

In line with our second hypothesis, we found that the analog home environment was related to language outcomes. We expected effects of the analog home environment to be especially visible regarding language skills. The analog home environment related to language in line with our expectations and previous work (e.g., Sénéchal et al., 1998), but not to literacy. Formal literacy activities have previously been shown to be related to language development (Sénéchal and LeFevre, 2002; Sénéchal, 2006). The questions that remained in the analog home environment factor after we ran the factor analysis, however, did not focus that much on formal literacy activities, as they mostly referred to storybook reading (see Table 2). Those questions that did focus on formal literacy activities had high loadings on both the digital and analog home environment factor and hence excluded from further analyses. More research is clearly needed to further understand this outcome.

In contrast to the second hypothesis, the digital literacy environment as measured in our study was not related to outcome measures regarding language and literacy or to a general measure such as non-verbal intelligence of the child. Similar results were found by Liebeskind et al. (2014) who found little effect of media on language skills of young children. It turns out that the fact that whether children play with language and word games on a computer or do letter naming games is not related to their language and literacy levels. The quality of the apps could very well have played an important role here, and it is a limitation of our study that we did not ask which apps were available at home. In an informal follow-up pilot, we did ask this question to parents of first-graders (Dimmendaal, 2018, unpublished). When asked which language and literacy apps their children played, YouTube was very often mentioned, as well as apps that are low in quality or, for example, use capital letter names instead of lower-case letter sounds. It is interesting to note that parents would mention YouTube as being a language or literacy app (see also Neumann and Herodotou, 2020). Clearly, more research is needed in this area. The results might suggest that the digital environment is rather omnipresent, while the analog home environment is a distinguishable factor in households regarding interest in language and literacy. If, for example, YouTube is used by a young child on a tablet (Neumann and Herodotou, 2020), the contents can have an endless variation in quality. This might suggest that it is not so much the quantity but the quality that matters regarding digital materials (see, e.g., Korat and Shamir, 2012). Also, the quality of the mediating role of the parent (Zaman et al., 2016) will have an impact on the effect of the DHLE. Indeed, we found low, and non-significant, correlations between early literacy skills and tablet use, similar to the results of Neumann (2016). Neumann did show a strong relation between print awareness and number of apps that parents reported and between tablet writing and both print awareness and print knowledge. We did not specifically ask about tablet writing or the number of apps on the tablet in our study. However, the number of devices at home or the number of digital books that were reported were not associated with children’s language or early literacy either, but only with the number of (paper) books in the home. The availability of materials at home is strongly related to SES and language development (Pace et al., 2017).

Our third hypothesis was that parental expectations would be related to language and early literacy, which would be partly mediated by both the analog and the digital home environment. Indeed, we found parental expectations to be related to both language and literacy, which is in line with results from Martini and Sénéchal (2012), for example. The relation was stronger for literacy, which can be ascribed to the fact that more questions were on literacy expectations than on language expectations. In contrast to previous research, we did not find any indirect effect of parental expectations. This might have a cultural reason, as in the Netherlands, home literacy activities are highly promoted, and parents in general are well aware of the importance of these (see, e.g., McElvany et al., 2012). Whether or not the parent has high expectations of the child would then be less related to how often, for example, the child is being read to at home. It is interesting to note that there was a positive correlation between parental expectations and the digital home environment; parents with higher expectations could be seen as the early adapters who create a more extensive digital literacy environment.

The above discussion already highlighted some limitations of the current study. First, we did not ask about the quality of the apps that were available in the home environment. Second, a substantial part of the sample did not fill out the questions on the digital home environment, and it is not clear why this was the case. In future research, it is recommended to interview the parents to gain more in-depth information about the digital home environment. It should be noted, though, that when we imputed the missing data, the results (both of the factor analyses and of the mediation analyses) remained the same. However, the sample size of the current study is relatively small, and results should therefore be interpreted with caution, also as participants were not the complete population of children in the participating schools, but only those that responded to the invitation. Third, while we asked about the educational level of the parents, research on the effects of the home literacy environment should take genetic factors more into account, by including direct measures of parental reading abilities (see, e.g., Puglisi et al., 2017; van Bergen et al., 2017). This will help to further understand the relation between children’s intelligence, parental education, and language and literacy outcomes. Fourth, we used questionnaires to assess the home literacy environment. Observations of the interactions between parents and children will give more insight in the quality of the home literacy environment, while ecological momentary assessments will give insight in real-time day-to-day activities. Finally, we acknowledge that the study had a cross-sectional design, so no causal claims can be made. Also, as we used a relatively small convenience sample, generalization of the results should be done with caution. A longitudinal study, in which the impact of both digital and analog home environment on the growth of language and literacy skills can be determined in a broad sample, is needed.

To conclude, we have shown that a DHLE can be distinguished from an analog (more traditional) home environment. The fact that there was no relation between the digital home environment and language and literacy outcomes suggest that there might be large variation in the quality of the digital home environment. More attention is needed to this part of the daily lives of children growing up in a digital society.

## Data Availability Statement

The datasets generated for this study are available on request to the corresponding author.

## Ethics Statement

Ethical review and approval was not required for the study on human participants in accordance with the local legislation and institutional requirements. Written informed consent to participate in this study was provided by the participants’ legal guardian/next of kin.

## Author Contributions

ES and TK contributed to conception and design of the study. ES organized the database. ES and TK performed the statistical analysis. ES wrote the first draft of the manuscript. TK wrote sections of the manuscript. Both authors contributed to manuscript revision, read and approved the submitted version.

### Conflict of Interest

The authors declare that the research was conducted in the absence of any commercial or financial relationships that could be construed as a potential conflict of interest.
